# Limited effects of non-steroidal anti-inflammatory drugs (NSAIDs) on imaging outcomes in osteoarthritis: observational data from the osteoarthritis initiative (OAI)

**DOI:** 10.1186/s12891-025-09172-z

**Published:** 2025-10-08

**Authors:** Virginie Kreutzinger, Katharina Ziegeler, Johanna Luitjens, Gabby B. Joseph, John Lynch, Nancy E. Lane, Charles E. McCulloch, Michael Nevitt, Thomas M. Link

**Affiliations:** 1 Department of Radiology and Biomedical Imaging, University of California - San Francisco, San Francisco, CA USA; 2 Department of Medicine, Center for Musculoskeletal Health, University of California - Davis, Sacramento, CA USA; 3 Department of Epidemiology and Biostatistics, University of California - San Francisco, San Francisco, CA USA

**Keywords:** Knee osteoarthritis, Magnetic resonance imaging, Imaging phenotypes, NSAID

## Abstract

**Background:**

Non-steroidal anti-inflammatory drugs (NSAIDs) are commonly prescribed for pain relief in osteoarthritis (OA), and their anti-inflammatory effects may play a role in shaping the disease course. The aim of this investigation was to examine the relationship between new use of prescription NSAIDs and changes in imaging biomarkers of synovitis in the knee, and to evaluate the association of NSAID use with structural cartilage damage over a period of four years.

**Methods:**

Applying a new user design to identify treatment effects in observational data, we selected participants from the Osteoarthritis Initiative (OAI) who were prescribed regular, oral NSAID medication between baseline and 48 months follow-up and who had available 3T MRIs of the right knee with whole-organ magnetic resonance imaging score (WORMS) readings as well as semi-quantitative assessments of synovitis for both timepoints. These individuals were frequency-matched with non-NSAID users matching for age, gender, body mass index (BMI), baseline Kellgren & Lawrence (KL) grade, Western Ontario and McMaster Universities Osteoarthritis (WOMAC) scores, and for the presence of an inflammatory imaging phenotype at baseline. Ordinal regression analyses and marginal estimated means were used to determine the effect of NSAID use on structural imaging outcomes, controlling for age, gender, BMI, and non-prescription NSAID use.

**Results:**

In this longitudinal analysis over 48 months, 142 individuals met prespecified criteria for new NSAID exposure, and 707 matched controls were identified. Regression analyses did not show a significant association between new NSAID use and changes in effusion-synovitis, Hoffa’s synovitis, or synovial proliferation scores over 4 years. However, NSAID users showed a significantly slower progression of cartilage lesions as measured by WORMS grading; this effect was marginally more pronounced in participants with an inflammatory imaging phenotype (beta − 0.92; *p* = 0.043) than in the population overall (beta − 0.48; *p* = 0.020).

**Conclusion:**

New NSAID use was not associated with MRI-detected synovitis over 4 years but had a modest association with reduced structural cartilage damage progression. This effect was more pronounced in individuals with an inflammatory imaging phenotype.

## Background

Osteoarthritis (OA) is a highly prevalent joint disease, causing considerable pain and disability [1, 2], with over 53 million affected individuals in the United States alone [3–5]. Recent advancements in the pathophysiological understanding of OA have led to a paradigm shift, recognizing the disease’s heterogeneity and the pivotal role of inflammatory processes in its progression [6–9]. This has been further elucidated through the development of OA phenotypes, which categorize the disease based on observable characteristics, including morphological changes detected through advanced imaging techniques [6, 10, 11]. The work of Romer et al., for example, has been instrumental in identifying distinct OA phenotypes on magnetic resonance imaging (MRI), such as variations in subchondral bone integrity, meniscus cartilage alterations, and an inflammatory phenotype characterized by synovitis [12–15]. The recognition of synovitis—a condition marked by the inflammation of the synovial membrane—as a critical factor in OA’s pathogenesis represents a significant advancement [7, 16]. This has shifted the focus towards understanding the molecular pathways that mediate inflammation and their contribution to the degradation of cartilage and subchondral bone [17, 18]. Current evidence suggests that inflammatory mediators, including cytokines and chemokines released by synovial inflammation, play a crucial role in the early stages of OA, potentially preceding and predicting the structural joint damage commonly associated with the disease [7].

This emerging understanding presents a compelling argument for the reevaluation of therapeutic strategies, specifically the potential of targeting inflammatory pathways as a means to modify disease progression [17, 19, 20]. The role of pharmacological agents, such as non-steroidal anti-inflammatory drugs (NSAIDs) and selective COX-2 inhibitors, in managing OA symptoms through their anti-inflammatory effects, offers a window into the disease-modifying potential of anti-inflammatory treatments [21, 22]. Previous studies on NSAID use in OA have yielded heterogeneous results: NSAID use has been associated with delayed knee replacement surgery by Higa et al. [22] and Lapane et al. showed modest symptom improvements in long-term NSAID users [23], while Simic et al. showed a worsening of radiographic outcomes in a randomized controlled trial of NSAID use [24]. As yet unaddressed is the question of whether there is a quantifiable effect of NSAID use on inflammation detected on MRI.

The aim of this investigation was to examine the relationship between new NSAID use and changes in imaging biomarkers of synovitis in the knee and to evaluate the effects of NSAID treatment on structural cartilage damage progression over a period of four years.

## Methods

### Participants and study design

Included in this study were participants from the observational multi-center study, the Osteoarthritis Initiative (OAI, https://nda.nih.gov/oai), a multi-center longitudinal, observational study involving 4,796 participants, focused on evaluating biomarkers in osteoarthritis. The dataset collected contained details about demographics, medication drug inventory, MR imaging of the right knee, and knee radiographs. Furthermore, the dataset incorporated the Western Ontario and McMaster Universities Osteoarthritis Index pain scale (WOMAC) [25] which has a maximum score of 20. This scale measures pain across five activities—surface walking, stair climbing, night-time awakenings, and periods spent sitting, lying, or standing—with each activity rated from 0 (no pain) to 4 (extreme pain).

**Fig. 1:**
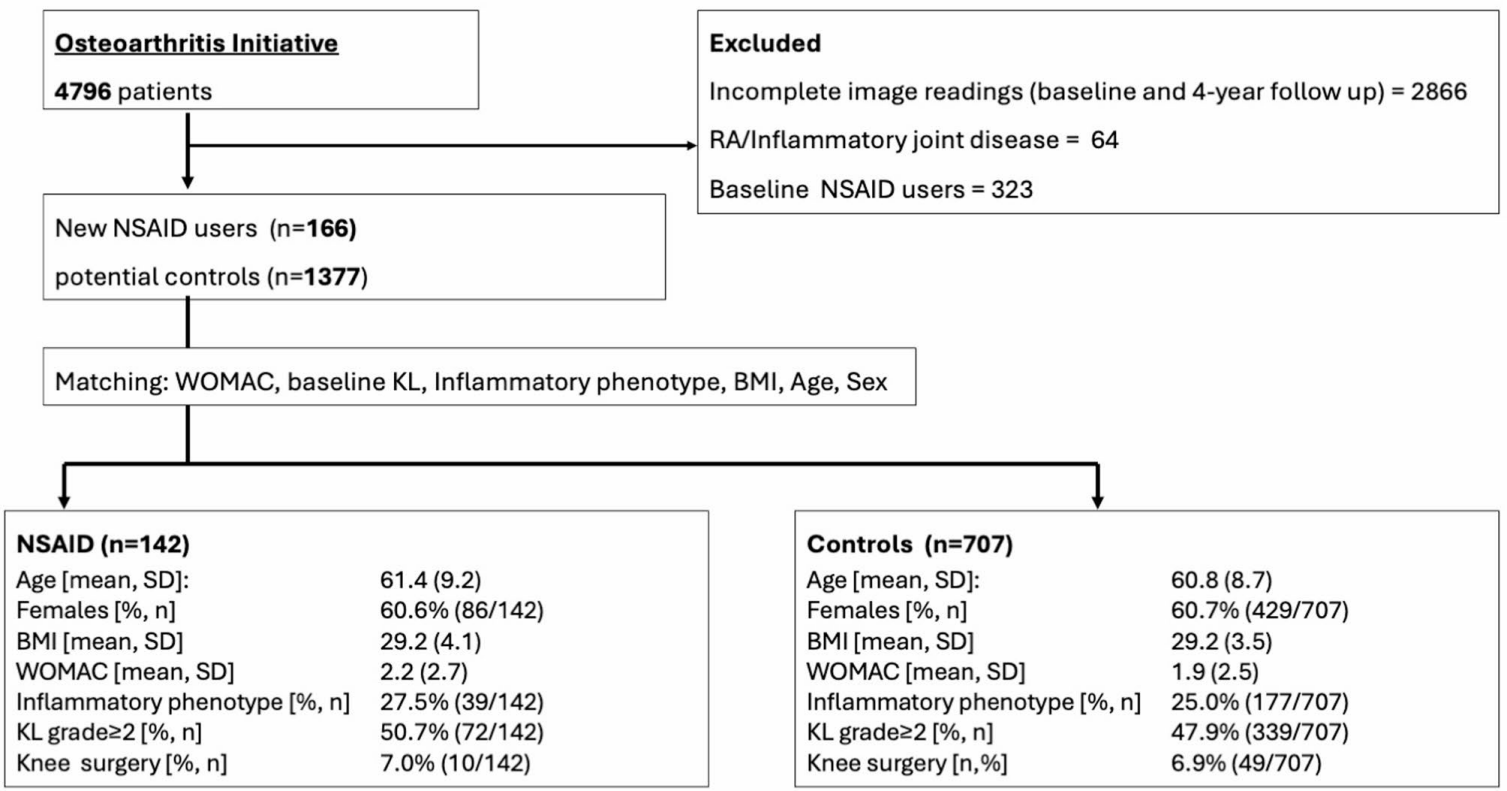
Participants selection and clinical characteristics. BMI = Body mass index (kg/m2). WOMAC = Western Ontario and McMaster Universities OsteoArthritis Index. KL = Kellgren & Lawrence

We included all participants with available semi-quantitative assessments of magnetic resonance imaging of the right knee at study enrollment and after 48 months. We excluded participants who stated in the questionnaire that they had rheumatoid arthritis or an inflammatory joint disease. A flowchart of participant inclusion, as well as demographic characteristics, is given in Fig. 1.

This investigation utilized a new user design [26] to simulate treatment effects in observational data, which is an established methodological approach [23]. Individuals with clearly defined NSAID exposure during the observational period (see section below) were matched with non-exposed controls at baseline for the following parameters: WOMAC at baseline, BMI at baseline, age at baseline, KL grade of the right knee at baseline, inflammatory imaging phenotype of the right knee and self-reported gender at baseline. The intended matching ratio was 6 controls for every case to maximize statistical power.

The study received approval from the institutional review boards (IRBs) at all OAI clinical sites and the coordinating center: the University of Pittsburgh Institutional Review Board, the Memorial Hospital of Rhode Island Institutional Review Board, the Committee on Human Research at the University of California, San Francisco, the Ohio State University’s Biomedical Sciences Institutional Review Board, and the University of Maryland Baltimore Institutional Review Board, (Approval Number: 10–00532). All participants provided written informed consent before participating. All investigations were carried out in compliance with the Declaration of Helsinki in its most current version.

### Definition of NSAID users

NSAID use was evaluated using the medical drug inventory from the Iowa Drug Information System, with specific drug codes ranging from 28,080,420 (tomeltin) to 28,080,764 (salsalate). This analysis included participants from the Osteoarthritis Initiative (OAI) who did not report NSAIDs use at the baseline visit and reported “regular” NSAID use for at least one year at any of the follow-up visits up to the 4-year visit. Subjects who used non-oral (e.g. topical) NSAIDs, took NSAIDs only for limited amounts of time, or only as-needed (rather than regularly) were excluded. Furthermore, questionnaire data on non-prescription NSAID use was collected and reported, but not used in the formal analysis, as it provided insufficient information on regularity of intake over extended periods of time.

### Imaging acquisition

All participants underwent a 3 T MRI of the knee at baseline and again after a 4-year follow-up through the OAI, using four identical MR scanners (Trio, Siemens Medical Solutions, Erlangen, Germany). The MRI protocol for the knee, which was previously published [27], included a coronal 2D intermediate-weighted (IW) turbo spin-echo (TSE) sequence, a sagittal fat-saturated 2D IW TSE sequence, and a sagittal 3D dual-echo steady-state sequence with water excitation (DESS WE). These sequences were used for semi-quantitative grading of knees. Detailed information about these sequences is documented in the OAI MR protocol (https://nda.nih.gov/oai/image-acquisations)..

### Image assessments

#### Synovitis and phenotype grading

**Fig. 2:**
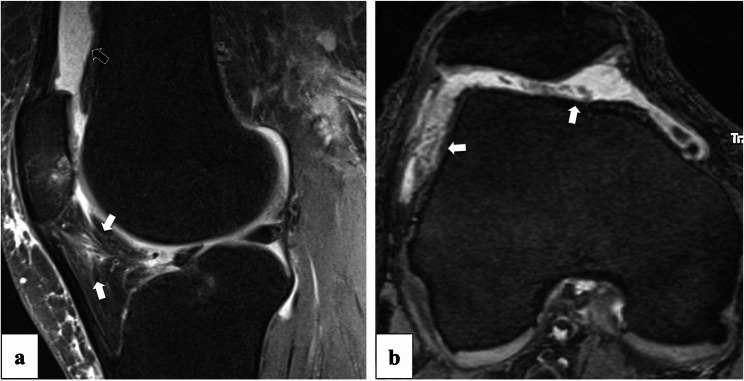
Image examples for an inflammatory phenotype. a = sagittal knee MRI with Hoffa synovitis (white arrows; Grade 2) and effusions synovitis (black arrow, Grade 3). b = axial knee MRI with moderate effusion synovitis (Grade 2) and extensive synovial proliferation (white arrows, Grade 3)

Images were semi-quantitatively evaluated for MR biomarkers of synovial inflammation using five distinct metrics: the Anterior Cruciate Ligament OsteoArthritis Score (ACLOAS) [28], the MRI Osteoarthritis Knee Score (MOAKS) [29], the size and signal intensity of infrapatellar fat pad (IFP) abnormalities, and the synovial proliferation score (SPS) [30]. ACLOAS scoring involved grading effusion-synovitis on sagittal fs IW images by measuring the suprapatellar recess’s maximum anteroposterior diameter using the midline section, with grades ranging from 0 (< 2 mm) to 3 (≥ 10 mm). MOAKS effusion-synovitis was assessed on axial fs IW images using a 4-point scale from 0 (physiological amount) to 3 (large with capsular distention). The size and signal intensity of IFP Hoffa synovitis ranged from 0 (no abnormality) to 3 (≥ 66% of the region affected). The intensity of IFP signal abnormalities was scored from 0 (normal) to 3 (similar to fluid intensity). For SPS, scores were modified to range from 0 (no effusion or effusion without synovitis) to 2 (extensive thickening with villonodular proliferation). Representative MR images for an inflammatory phenotype are provided in Fig. 2. We used a modified Rapid Osteoarthritis MRI Eligibility Score (ROAMES) to better account for early disease and to classify participants as having an inflammatory phenotype. The original ROAMES [13] system distinguishes between five different phenotypes: inflammatory, meniscus/cartilage, subchondral bone, atrophic, and hypertrophic. However, for this study, we focused exclusively on the inflammatory phenotype, due to the proposed anti-inflammatory mechanism of action of NSAID. To meet the criteria for an inflammatory phenotype, participants needed to exhibit a combination of at least mild suprapatellar joint effusion (MOAKS effusion synovitis score ≥ 1) and irregularity of the synovial membrane (SPS score ≥ 1).

#### WORMS grading

The images were evaluated for OA features by using the UCSF modified semiquantitative WORMS grading system [31–33]; for the purpose of this analysis, only the cartilage and bone marrow lesion gradings were used. Cartilage defects were scored from 0 to 6, and bone marrow edema like lesions (BMELLs) were scored from 0 to 3 in the same six regions (patella, trochlea, medial femur, lateral femur, medial tibia, and lateral tibia). For the WORMS cartilage subscale a max score was calculated, while for the BMELL subscale a sum score was calculated [34].

#### Statistical analysis

All statistical analyses were conducted using SPSS Version 29. Separate ordinal regression analyses were performed, with change effusion synovitis, Hoffa’s synovitis, synovial proliferation, WORMSs cartilage and BMELL scores as outcomes and prescription NSAID use as predictor; these regression models were adjusted for age, self-reported gender, BMI at baseline, and use of non-prescription NSAIDs at any timepoint during the observation period. Furthermore, adjusted marginal means with the same covariates were computed for baseline and 4-year follow-up. All analyses were performed for the whole study population and separately for the subgroup of participants with an inflammatory imaging phenotype, to simulate recruitment stratification by imaging phenotype. For significant effects, an interaction analysis for inflammatory imaging phenotype was performed to test whether differences in effect were statistically significant. The significance level for all statistical tests was set at an alpha of 0.05.

## Results

### Participants

A total of 142 NSAID users and 707 matched controls were identified. Basic demographics of the included participants can be found in Fig. 1. NSAID use was initiated at 12-month follow-up in 75 users, at 24-month follow-up in 28 users and 36-month follow-up in 39 users. Per our study design, there were no significant differences between NSAID users and controls regarding age (61.4±8.7 years in NSAID users vs. 60.8±8.7 years in controls; *p* = 0.406), gender (60.6% vs. 60.7% women; *p* = 0.979), BMI (29.2±4.1 vs. 29.2±3.5; *p* = 0.910), KL grade at baseline (KL ≥ 2 50.7% vs. 47.9%; *p* = 0.576), inflammatory phenotype at baseline (27.5% vs. 25.0%; *p* = 0.544), and WOMAC pain at baseline (2.2±2.7 vs. 1.9±2.5; *p* = 0.149). Rates of knee replacement (right side), as detected on follow-up, were low in both groups (7.0% vs. 6.9%; *p* = 0.652). Concomitant use of non-prescription NSAIDs as well as acetaminophen were more common in (prescription) NSAID users than controls. Acetaminophen use was reported by 35.2% (50/142) of NSAID users vs. 22.5% (159/707 controls) (*p* = 0.001) and non-prescription NSAID use was reported by 50.7% (72/142) of prescription NSAID users and 39.3% (278/707) controls (*p* = 0.012).

### Synovitis

At baseline, future NSAID users had similar gradings for effusion synovitis measured by ACLOAS and Hoffa’s synovitis as controls (see Table 1), while controls had higher baseline effusion synovitis scores measured by MOAKS. Synovial proliferation scores (range 0–2) at baseline, were marginally higher in future NSAID users than controls (0.38 vs. 0.28), but the difference missed statistical significance (*p* = 0.055). In the subgroup of participants with an inflammatory imaging phenotype, mean scores of all markers of synovitis were higher than in the cohort as a whole.

**Table 1:** Imaging outcomes – baseline group comparisons and change over 48 months. 95% CI = 95% confidence interval. Coefficients show association of NSAID use and respective imaging outcome, from ordinal regression analyses, adjusted for gender, age, BMI at baseline, and use of non-prescription nsaids. Significantly higher values (*p* < 0.05) are printed in bold

			baseline				Change over 48 months			
			mean	95%CI		p	coeff.	95%CI		p
				lower	higher			lower	higher	
All participants	ACLOAS effusion	Controls	0.72	0.67	0.78	0.819	0.03	-0.35	0.40	0.888
		NSAID	0.71	0.58	0.83					
	MOAKS effusion	Controls	1.06	1.01	1.11	0.035	0.04	-0.34	0.43	0.832
		NSAID	0.93	0.82	1.04					
	Hoffa’s size	Controls	0.98	0.94	1.03	0.332	0.36	-0.05	0.14	0.089
		NSAID	0.93	0.82	1.03					
	Synovial proliferation	Controls	0.29	0.26	0.33	0.055	0.38	-0.05	0.82	0.081
		NSAID	0.38	0.29	0.47					
	WORMS cartilage	Controls	3.25	3.12	3.37	0.071	-0.48	-0.88	-0.08	0.020
		NSAID	3.52	3.25	3.80					
	WORMS BMELL	Controls	2.18	2.03	2.33	0.964	0.03	-0.31	0.37	0.870
		NSAID	2.17	1.82	2.52					
Inflammatory phenotype	ACLOAS effusion	Controls	1.23	1.12	1.35	0.136	−0.27	−0.97	0.44	0.454
		NSAID	1.02	0.78	1.27					
	MOAKS effusion	Controls	1.56	1.47	1.66	0.598	0.16	−0.54	0.85	0.657
		NSAID	1.50	1.30	1.70					
	Hoffa’s size	Controls	1.26	1.17	1.36	0.282	−0.26	−1.15	0.63	0.570
		NSAID	1.14	0.93	1.35					
	Synovial proliferation	Controls	1.13	1.07	1.18	0.018	0.06	−0.79	0.92	0.883
		NSAID	1.28	1.16	1.39					
	WORMS cartilage	Controls	3.78	3.56	4.01	0.049	−0.92	−1.80	−0.03	0.043
		NSAID	4.32	3.84	4.80					
	WORMS BMELL	Controls	2.64	2.30	2.98	0.569	−0.32	−0.98	0.34	0.341
		NSAID	2.88	2.15	3.61					

In this subgroup, baseline synovial proliferation scores of future NSAID users were also higher than those of controls (1.28 vs. 1.13; *p* = 0.018). Change of these markers over 48 months was investigated with ordinal regression analyses, results of which are also given in Table 1. None of the imaging markers of synovitis was significantly impacted by NSAID use over 48 months, neither in the study population as a whole nor in the subset of participants with an inflammatory imaging phenotype (Table 1).

### Cartilage defects

**Fig. 3:**
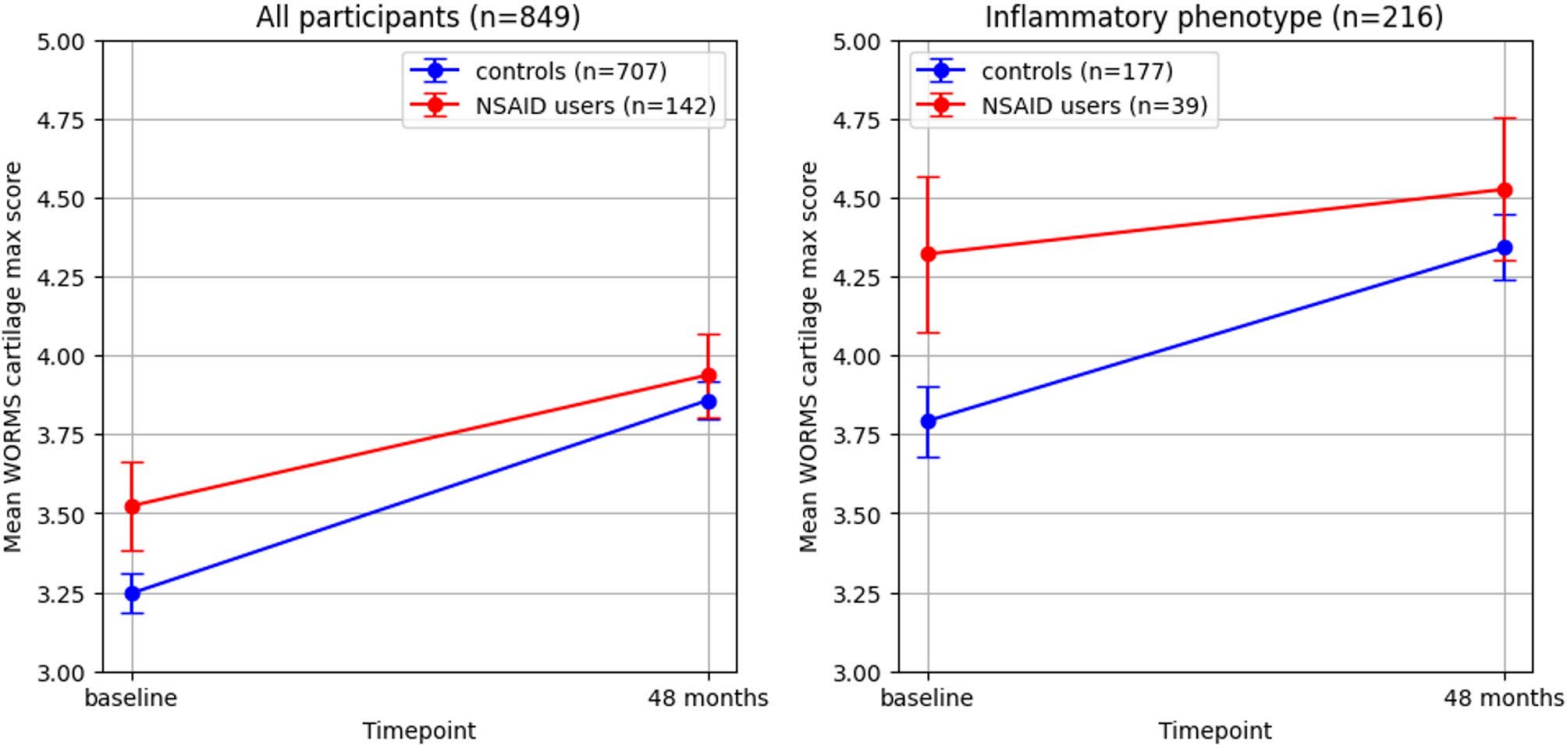
Progression of cartilage maximum scores over 4 years, adjusted means. Left: all participants; right: inflammatory phenotype only. X-axis: time points. Y-axis: mean change in WORMS cartilage maximum score (scale adjusted; actual score range 0–6). Note the less steep increase in NSAID users, which is more pronounced in the inflammatory phenotype

Adjusted maximum cartilage lesion grade (on a scale from 0 to 6) at baseline was 3.52 in NSAID users and 3.25 in controls, the difference was not statistically significant (*p* = 0.071); these scores were higher in participants with an inflammatory phenotype (4.32 in NSAID users vs. 3.78 in controls; *p* = 0.049). The association of new NSAID use with change in these scores over 48 months was investigated using ordinal regressions, adjusted for age, gender, BMI at baseline and use of non-prescription NSAIDs; results are given in Table 1, and a graphical representation of change in adjusted mean cartilage scores over time is provided in Fig. 3. Results of these analyses indicate a small but significant association of NSAID use and reduced cartilage lesion progression with a beta of −0.48 (95%CI −0.88– −0.08; *p* = 0.020) and a visibly less steep increase of cartilage scores in NSAID users. These effects were more pronounced in participants with an inflammatory imaging phenotype at baseline, with a larger beta of −0.92 (95%CI −1.80– −0.03; *p* = 0.043). A formal interaction analysis showed that these differences were not statistically significant, however (*p* = 0.361).

### Bone marrow edema like lesions

The extent of BMELLs at baseline, expressed as a sum score (ranging from 0 to 18) did not differ significantly (*p* = 0.964) between NSAID users (2.17) and controls (2.18) at baseline; the same held true in the subset of participants with an inflammatory imaging phenotype (NSAID: 2.88; controls: 2.64; *p* = 0.569) (see also Table 1). Change in BMELL max score over 48 months, which was analyzed by ordinal regression, was not significantly impacted by NSAID use (beta: 0.03; *p* = 0.870). The same observation was made in the subgroup of participants with an inflammatory phenotype (beta: −0.32; *p* = 0.341) (see Table 1).

## Discussion

NSAIDs are among the most widely used pharmacological interventions in osteoarthritis, although evidence for structural disease modulation is sparse. Our study is the first to investigate the effect of new regular oral NSAID use on MR imaging outcomes in osteoarthritis, while at the same time attempting to investigate the effect specifically in participants with an inflammatory imaging phenotype. In this group of NSAID users and closely matched controls, we found small but significant positive effects of regular NSAID use on the progression of structural cartilage damage but no discernible effects on imaging markers of synovitis.

The notion of stratifying patients based on imaging characteristics/phenotypes of osteoarthritis in the context of clinical trials has gained traction in recent years [16, 35, 36]. In our study, which focused primarily on the supposed anti-inflammatory effect of NSAIDs, we chose to separate out individuals with signs of active inflammation on imaging, i.e. those fulfilling criteria of an inflammatory phenotype. While the specific criteria used to determine this phenotype are still the topic of scientific debate [13, 37], there is a consensus, that patients with dominant synovitis represent a distinct subgroup of OA [38]. While our data showed marginal differences in response to NSAID treatment between individuals with an inflammatory phenotype vs. without, we were not able to establish statistical significance for these group differences.

NSAIDs exert their anti-inflammatory effects through the inhibition of cyclooxygenase (COX), which is involved in the synthesis of proinflammatory cytokines, most prominently prostaglandins [39]. Furthermore, recent studies have demonstrated a decrease of Interleukin 6 (IL-6), tumor necrosis factor alpha (TNF-alpha) and vascular endothelial growth factor (VEGF) in the synovial fluid of OA patients on NSAID therapy [20]. Based on this, we hypothesized, that NSAID treatment should have a favorable effect on synovitis on imaging, which could, however, not be confirmed in our analysis. Apart from a possible true lack of anti-inflammatory effects of NSAID therapy, these findings may at least in part be explained by the methods deployed to quantify inflammation. The two most established markers for the detection quantification of joint inflammation are the detection of joint effusion [28, 29] and increases in the fluid signal of Hoffa’s fat pad. Neither of these markers decreased with NSAID therapy in our study population. In clinical reality, however, synovial inflammation in OA is often undulating rather than linearly progressive, making the longitudinal measurement of this condition more vulnerable to sampling error. Thus, rather than focusing on joint effusion, formal assessment joint inflammation often relies on changes in morphology and perfusion of the synovial membrane [40]. Translating this concept to non-contrast enhanced joint imaging, the thickening of the synovial membrane, measured by the synovial proliferation score [30], was hypothesized to better demonstrate sustained or recurrent synovial inflammation. Our results also did not show an effect of NSAID treatment in this imaging biomarker, however. We therefore conclude that the anti-inflammatory effects of NSAIDs are not potent enough, to affect this imaging biomarker.

While synovitis is associated with pain and clinical symptoms in OA [41] and sustained synovitis has been tied to structural disease progression [30] on imaging, the outcome measure of greater significance in OA is the progression of cartilage damage. In our study population, we saw a protective effect of NSAID treatment on structural cartilage damage. This ties in with the findings of Lapane et al. who demonstrated a modest slowing of medial femorotibial joint space narrowing in NSAID users [23]. Interestingly, the effect was slightly more pronounced in participants with an inflammatory imaging phenotype on baseline imaging. This might have been caused by a decrease in pro-inflammatory cytokines under NSAID therapy [20], even if the effect was not strong enough to translate to a decrease in synovial inflammation on imaging.

As one of the imaging lesions with a close relationship to pain [42] and one of the earlier markers for structural progression [43], the effect of NSAIDs on BMELLs was a secondary aim of this analysis. Current therapeutical approaches targeting BMELLs in OA focus less on inflammatory pathways and rather on bone metabolism [36], mainly by using therapeutic agents developed for the treatment of osteoporosis [44]. Even these more targeted agents show modest effects at best – therefore it is not surprising, that we were not able to show an effect of NSAID use on the change of BMELLs over 4 years.

Our study has several limitations, that warrant critical discussion. First and foremost, observational data inherently has severe limitations in demonstrating treatment effects, and findings from our investigation are not to be interpreted in the same way that data from a randomized controlled trail could be. Despite attempting to control for disease severity at baseline, deploying an established methodology for observational data [23, 26], the fact that participants reported regular NSAID use may in itself be an indicator that these were individuals with a more severe course of OA than their respective controls. Despite applying a matching strategy, that may have resulted in residual confounding, which may have been avoided with a more rigorous propensity score matching, we had a limited the pool of eligible participants, which in turn negatively impacted the statistical power of this investigation. This limitation is further translated into the fact that we were not able to conduct further analyses on different user groups (e.g. different active components or formulations) or otherwise clinically meaningful subgroups. Lastly, as imaging was not available for all timepoints and some participants initiated NSAID treatment later during the observational period, there was potential for significant delay between initial assessment of imaging changes and start of NSAID use, which may have introduced a bias and an underestimation of actual treatment effects.

## Conclusion

In summary, we found a modest deceleration of the progression of structural cartilage damage in new regular NSAID users, which was marginally more pronounced in participants with an inflammatory imaging phenotype, although no statistically significant difference of effects between both groups could be established. Interestingly, we did not find a significant association between NSAID use and imaging biomarkers of synovial inflammation. These findings underscore the importance of including different joint tissues as imaging outcomes in OA, regardless of proposed drug mechanism, given the complex multi-tissue nature of the disease and the limitations of capturing this complexity using imaging alone. They furthermore highlight the need for continued work on improving methods for detection and quantification of joint inflammation in OA.

## Data Availability

The underlying data is publicly available (OAI, https://nda.nih.gov/oai).

## Abbreviations

3 T: 3 Tesla
ACLOAS: Anterior cruciate ligament osteoarthritis score
BMI: Body mass index
BMELLs: Bone marrow edema like lesions
COX-2: Cyclooxygenase-2
DESS WE: Dual-echo steady-state sequence with water excitation
IFP: Infrapatellar fat pad
IW: intermediate-weighted
KL: Kellgren Lawrence
MOAKS: MRI Osteoarthritis Knee Score
NSAIDs: Non-steroidal anti-inflammatory drugs
OA: Osteoarthritis
OAI: Osteoarthritis initiative
ROAMES: Rapid osteoarthritis MRI eligibility score
SPS: Synovial proliferation score
TNF-alpha: Tumor necrosis factor
TSE: Turbo spin echo
VEGF: Vascular endothelial growth factor
WOMAC: Western ontario mcmaster universities osteoarthritis
WORMS: Whole-organ magnetic resonance imaging score
